# Editorial: Organ mimicking technologies and their applications in drug discovery

**DOI:** 10.3389/fbioe.2023.1341153

**Published:** 2023-12-01

**Authors:** Xiuli Zhang, Yong Luo, Qun Wang

**Affiliations:** ^1^ Jiangsu Key Laboratory of Neuropsychiatric Diseases and College of Pharmaceutical Sciences, Soochow University, Suzhou, Jiangsu, China; ^2^ State Key Laboratory of Fine Chemicals, Department of Pharmaceutical Engineering, School of Chemical Engineering, Dalian University of Technology, Dalian, Liaoning, China; ^3^ Department of Chemical and Biological Engineering, Iowa State University, Ames, IA, United States

**Keywords:** organ-on-a-chip, organoid, 3D bioprinting, nonclinical tests, artificial organs

The drug discovery industry has gradually recognized the importance of biomimetic evaluation methods in nonclinical tests, particularly with the recent introduction of the FDA Modernization Act 2.0 (Ahmed et al., 2023). The development of artificial organs that can faithfully recapitulate the function of real organs has taken center stage in this field. Various technologies, such as organ-on-a-chip (Leung et al., 2022), organoid (Kim et al., 2020; Zhao et al., 2022), and 3D bioprinting (Murphy and Atala, 2014), are utilized to construct these artificial organs.

Organ-on-a-chip is an advanced cell or microtissue co-culture system that utilizes precise 3D localization, advanced biomaterials, and microfluidics to replicate physiological conditions. Its power lies in its ability to customize the microenvironments of cells or microtissues. Numerous physical and chemical cues that control the growth and performance of cells or microtissues can be recreated in the microfluidic device, such as shear force, concentration gradient, and paracrine effects. Additionally, it allows for real-time monitoring and analysis of cellular and molecular changes, enabling researchers to observe drug responses and disease progression directly. The research works by (Qu et al., 2018), and Jiu Deng et al. (Deng et al., 2019a; Deng et al., 2019c; Deng et al., 2020) sufficiently reflect the power of organ-on-a-chip technologies. Recently, the research focus has shifted to the construction of organ-on-a-chip using stem cells (Musah et al., 2017) or organoids (Ronaldson-Bouchard et al., 2022), and the traditional cell-line or primary cell-based organ-on-a-chip systems have moved towards commercialization. Many companies are involved in this field, including Emulate and Mimetas.

An organoid is a self-organized 3D microtissue typically derived from stem cells (pluripotent, fetal or adult), mimicking an organ’s essential functional, structural and biological complexity (Zhao et al., 2022). Various organoids have been reported, including the brain (Bang et al., 2021), liver (Deng et al., 2019b), kidney (Homan et al., 2019), pancreas (Shik Mun et al., 2019), and intestine (Puschhof et al., 2021; Tong et al., 2023), blood vessel (Wimmer et al., 2019). This burgeoning technology is still in its early stages. Current organoids primarily mimic embryonic or infant organs, necessitating the development of organoids that can simulate adult organs. Although there have been reports on the vascularization of organoids (Homan et al., 2019), the overall level of vascularization still requires improvement. Existing organoid technologies also have rarely addressed aspects such as the immune system. Although there are many limitations, organoids are favored in the scientific communities, primarily because all the cells in organoids are derived from humans.

3D bioprinting is an additive manufacturing technology that utilizes 3D printing to create active, three-dimensional structures using cells enclosed in biological materials. The ultimate objective of 3D bioprinting is the construction of transplantable organs, making the 3D bioprinted organs suitable for drug screening. However, the main challenge for drug discovery applications lies in the size of these 3D bio-printed organs, which is more significant than organs-on-a-chip and organoids. This limitation restricts their use for large-scale screening. Nevertheless, the increasing resolution of 3D bioprinting is expected to alleviate this situation (He et al., 2023a).

**FIGURE 1:**
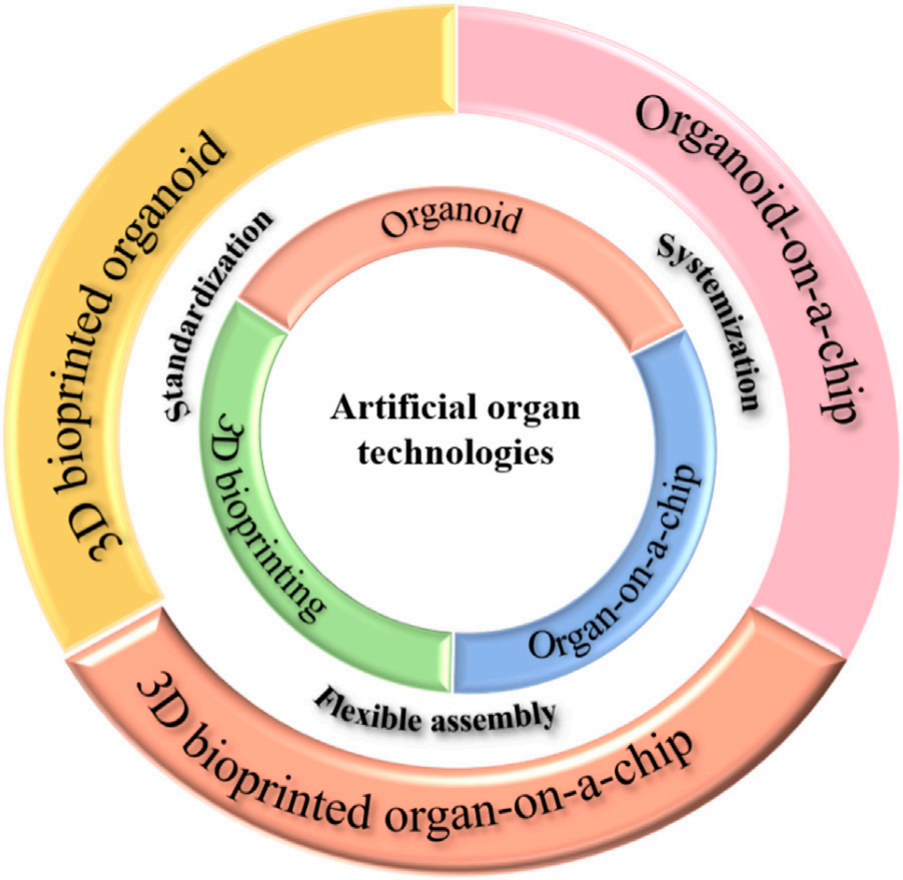
The artificial organ technologies system.

By combining these three technologies and incorporating hyphenation, it is possible to obtain more biomimetic models. Summarily, using 3D bioprinted organ chips (Rahmani Dabbagh et al., 2023) allows for greater flexibility in construction and feasibility. 3D bioprinted organoids (Brassard et al., 2021) streamline and automate the process of organoid construction. The organoid chip (Park et al., 2019) facilitates vascularization and the examination of interaction between multiple organs. As research in this field (Figure 1) continues to thrive, a future in healthcare may emerge where personalized medicine and revolutionary treatments become the standard, alongside advancements in drug discovery.

To summarize the recent advancements of organoid technology and applications in lung diseases, Chen and Na discussed the applications of lung organoids in the studies of various lung diseases, such as lung cancer, influenza, cystic fibrosis, idiopathic pulmonary fibrosis, and the recent severe acute respiratory syndrome coronavirus-2 (SARS-CoV-2) pandemic (Chen and Na). They also provided an update on the generation of organoid models for these lung diseases and their applications in basic and translational research, highlighting these signs of progress in pathogenesis study, drug screening, personalized medicine and immunotherapy. Furthermore, they discussed the current limitations and future perspectives in organoid models of lung diseases (Chen and Na). Pereira et al. also shared their views on recent advances in organoid models on neurodegenerative diseases (Pereira et al.). Many neurodegenerative diseases are identified, but their causes and cure are far from understood because of the complexity of the neural tissue and its challenges for evaluation. To solve these problems, they reviewed the latest *in vitro* models used to study neurodegenerative disease and how they have evolved by introducing microfluidics platforms, 3D cell cultures, and induced pluripotent cells to mimic the neural tissue environment in pathological conditions (Pereira et al.). To integrate immune cells in organs-on-chip models to the next level and enable them to mimic complex biological responses, Van Os et al. summarized blocks of an organs-on-chip model of acute infection to investigate the recruitment of circulating immune cells into the infected tissue. The multi-step extravasation cascade *in vivo* is described, followed by an in-depth guide on how to model this process on a chip (Van Os et al.). The review focuses on the hydrogel extracellular matrix to accurately model the interstitial space through which extravasated immune cells migrate toward the site of infection. This tutorial review is a practical guide for developing an organs-on-chip model of immune cell migration from the blood into the interstitial space during infection (Van Os et al.).

In this Research Topic, other groups shared their most recent research results on using organoid platforms for drug discovery and development. Peng’s group established a living biobank of organoids derived from colorectal cancer (CRC) patients (He et al.). It explored the application prospect of patient-derived organoids (PDOs) in CRC as a preclinical model of precision cancer medicine. They also evaluated whether CRC PDOs could effectively predict patient drug response in clinical practice. Ong et al. have performed a comparative study of tumor-on-chip models with patient-derived xenografts (PDX) for predicting chemotherapy efficacy in colorectal cancer patients. Indeed, this is the first case of a direct in vitro-in vivo comparative study to develop a comparative framework for drug response predictions made from tumor organ-on-a-chip models against those that matched PDX models in a patient-specific manner (Ong et al.). At last, Su et al. established retinal organoids (ROs) and microfluidic chip-based approaches to explore retinitis pigmentosa with USH2A mutations (Su et al.). To elucidate the molecular mechanism of retinitis pigmentosa (RP), transcriptomic and proteomic analyses were performed to identify significantly regulated genes and proteins related to USH2A mutations. They also constructed a microfluidic chip to co-culture ROs and retinal pigment epithelium (RPE) cells with USH2A mutations as an advancement to current organoid models (Su et al.).

We hope this Research Topic will give the audience an recent view of organ-on-a-chip and organoid technologies and their applications in drug discovery and development.
